# Peroxydisulfate Activation by Lignosulfonate-Derived Iron–Carbon Catalyst for Tetracycline Hydrochloride Removal: Contributions of ^1^O_2_ and Iron Cycle

**DOI:** 10.3390/toxics14070606

**Published:** 2026-07-11

**Authors:** Chun Xiao, Jinxi Chen, Yin Yang, Wu Ren, Lihong Ai, Yue Lu, Hongjun Li, Jiahui Zhang, Jiangfei Cao

**Affiliations:** 1 College of Environmental and Chemical Engineering, Zhaoqing University, Zhaoqing 526061, China; 13413944153@163.com (J.C.); 15523920456@163.com (Y.Y.); 18983325969@163.com (W.R.); 19923672850@163.com (L.A.); 13825395373@163.com (Y.L.); 15819387546@163.com (H.L.); 13729518245@163.com (J.Z.); 2 New Energy and New Materials Research Center, Zhaoqing University, Zhaoqing 526061, China

**Keywords:** iron–carbon catalyst, tetracycline hydrochloride, PDS activation, ^1^O_2_, non-radical pathway

## Abstract

A lignosulfonate-derived iron–carbon composite catalyst was fabricated via hydrothermal pyrolysis and employed to activate peroxydisulfate (PDS) for tetracycline hydrochloride (TCH) degradation. The optimized LFC possessed a porous carbon matrix uniformly decorated with Fe^0^/Fe_3_O_4_/Fe_2_O_3_ crystals, providing abundant active sites for catalytic reactions. The LFC/PDS system achieved nearly 100% TCH removal within 30 min at neutral pH and exhibited high efficiency over a broad pH range, strong anti-interference ability, and good universality for various organic pollutants. Mechanistic investigation confirmed that TCH degradation was dominated by a singlet oxygen (^1^O_2_)-mediated non-radical pathway, with minor contribution from radical species. The synergistic effect of iron cycle and surface functional groups promoted the generation of reactive oxygen species and ^1^O_2_. This research provides a low-cost, eco-friendly and efficient strategy for antibiotic wastewater treatment.

## 1. Introduction

Tetracycline hydrochloride (TCH), a widely used broad-spectrum antibiotic, has emerged as the second-largest class of antibiotics globally [1,2]. It is extensively employed in diverse fields such as medical treatment and animal husbandry [3]. However, the large-scale production and application of TCH have led to severe environmental residue issues, particularly water pollution, which poses a substantial threat to ecological systems and human health [4,5]. Moreover, due to its poor biodegradability and long-term persistence in aquatic environments [6], TCH cannot be efficiently eliminated by conventional treatment methods, thus making the development of novel, high-efficiency and eco-friendly wastewater treatment technologies an imperative priority [7,8]. Current removal technologies for TCH mainly include adsorption, photo-catalytic oxidation, microbial treatment and Fenton processes, yet all these approaches are plagued by inherent limitations. Specifically, adsorption merely transfers the contaminants rather than achieving their degradation, and it struggles with the treatment of high-concentration antibiotic wastewater [9]. Microbial degradation is characterized by low efficiency, along with the challenges of difficult and time-consuming strain cultivation [10,11]; more importantly, a major drawback of this method is its high tendency to induce the generation and dissemination of antibiotic resistance genes (ARGs). These ARGs can be horizontally transferred among various microbial communities in aquatic environments, leading to the emergence of antibiotic-resistant bacteria, which further reduces the efficacy of antibiotics in clinical and environmental settings and poses a potential threat to ecological balance and public health [12]. Traditional chemical oxidation methods exhibit limited oxidizing capacity, resulting in low TCH removal rates and only partial degradation of the antibiotic; notably, some of the resulting intermediates remain toxic and even exhibit higher toxicity than the parent compound, precluding complete mineralization.

In recent years, persulfate-based advanced oxidation processes (PS-AOPs), as an emerging technology, have become a research focus for industrial wastewater treatment owing to their unique merits, including high stability, fast reaction kinetics, convenient transportation and storage, and a wide applicable pH range [13,14]. The core of PS-AOPs is the activation of persulfate to generate highly oxidative reactive species (SO_4_^−^· and ·OH), which can efficiently degrade refractory organic pollutants such as antibiotics in aqueous environments [15,16,17].

Among various catalysts, iron-based materials have become a research hotspot in TCH degradation via AOPs due to their low cost and superior catalytic activity. Nevertheless, the non-selective oxidation behavior of such systems leads to significant performance deterioration in complex water matrices containing diverse competitive constituents, which severely restricts the practical application potential of this technology. Accordingly, there is an urgent need for novel technologies that can achieve efficient and selective removal of emerging pollutants in complicated water matrices. Meanwhile, iron can undergo valence cycling between Fe^2+^ and Fe^3+^. Through electron transfer reactions, it breaks the O-O bond in persulfate to generate strongly oxidative sulfate radicals (SO_4_^−^·) and hydroxyl radicals (·OH), thereby achieving efficient degradation of organic pollutants [18]. More importantly, some studies have demonstrated that carbon-supported iron can promote the targeted generation of non-radical ^1^O_2_ in PS-AOPs. Wang et al. prepared an iron-based biochar catalyst using peanut shell as a biomass carbon source via FeCl_3_ impregnation followed by high-temperature pyrolysis and carbonization. The carbon modification during pyrolysis endows the biochar with abundant defects and active functional groups, which can act as electron transfer mediators to regulate the redox cycle of iron species, thereby promoting the selective generation of ^1^O_2_ during persulfate activation; characterizations revealed that the catalyst efficiently degraded antibiotic pollutants mainly through a ^1^O_2_-dominated non-radical pathway [19]. Yang et al. fabricated magnetic waste tea biochar by modifying waste tea and loading magnetic components. The carbon modification of waste tea enhances the interfacial interaction between the carbon support and iron species, facilitating electron transfer and persulfate activation to generate ^1^O_2_, where the interfacial synergy further improves the activation efficiency of peroxymonosulfate [20].

Lignin is a natural polymer that is rich in aromatic rings; sodium lignosulfonate (SLS) is a derivative of lignin. SLS is also abundant, renewable, chemically stable, and low-cost. The structure of SLS includes many functional groups, such as alcohol hydroxyl groups, sulfonate, methoxy groups, and phenolic hydroxyl groups. These give it good chemical reactivity. SLS is often treated as a waste product in the paper industry. Therefore, using SLS to prepare carbon-modified iron-based catalysts is helpful to avoid environmental pollution and reduce the discharge of solid waste, which belongs to the concept of clean production.

Herein, using sodium lignosulfonate as the carbon matrix, a structurally stable and environmentally friendly iron–carbon composite catalyst (Lignosulfonate-Fe Carbonized Catalyst, LFC) was fabricated via hydrothermal synthesis by adjusting the iron salt dosage, hydrothermal temperature, and reaction time. Using TCH as the target pollutant, the degradation performance of TCH by LFC-activated peroxydisulfate (PDS) was systematically investigated. The effects of different reaction parameters on TCH removal efficiency were explored, and the mechanism of LFC-activated PDS for TCH degradation was elucidated. This work aims to provide a theoretical basis and technical reference for the application of iron–carbon-based catalyst/persulfate systems in antibiotic wastewater treatment.

## 2. Materials and Methods

### 2.1. Chemical

Ferric chloride hexahydrate (FeCl_3_·6H_2_O, analytical grade), ferrous chloride tetrahydrate (FeCl_3_·4H_2_O, analytical grade), oxalic acid (H_2_C_2_O_4_, analytical grade), tetracycline hydrochloride (TCH, analytical grade), and peroxydisulfate (PDS, K_2_S_2_O_8_, analytical grade) were all purchased from Guangzhou Chemical Reagent Factory (Guangzhou, Guangdong, China). Anhydrous ethanol (analytical grade) was supplied by Xilong Chemical Co., Ltd. (Shantou, Guangdong, China). Methanol (MeOH) and acetonitrile were of chromatographic grade and purchased from Siyou Chemical Reagent Co., Ltd., Tianjin (Tianjin, China). Furfuryl alcohol (FFA), tert-butanol (TBA), p-benzoquinone (BQ), Rhodamine B (RhB), Methylene Blue (MB), Sulfamethoxazole (SMX) and Phenol (PN) were all analytical grade products purchased from Sinopharm Chemical Reagent Co., Ltd. (Shanghai, China). Deionized water was used throughout the experiments.

### 2.2. Preparation of LFC

Dissolve 5.0 g SLS and 15.0 g FeCl_3_·6H_2_O in 50 mL deionized water, stir mechanically for 30 min, put it into a hydrothermal reactor, pyrolyze in a 160 °C oven for 4 h, then alternately wash 5 times with pure water and ethanol, centrifuge, and vacuum dry at 80 °C for 6 h to obtain the Lignosulfonate-Fe Carbonized Catalyst (LFC). In order to optimize the preparation conditions of LFC, this study considered the effects of the dosage of FeCl_3_·6H_2_O (10.0 g, 15.0 g and 20.0 g), reaction time (4 h, 6 h and 10 h) and reaction temperature (160 °C, 180 °C and 200 °C) on the product.

### 2.3. Catalytic Degradation Experiment

A total of 100 mL of the 50 mg/L TCH solution was transferred into a 250 mL Erlenmeyer flask. Following the addition of LFC, the suspension was ultrasonicated for 5 min to ensure homogeneity. The reaction was then initiated by the addition of PDS. An Erlenmeyer flask was placed in into a water bath constant temperature oscillator at room temperature and oscillated at a oscillation speed of 180 r/min. In total, 1 mL of the solution was filtered with a 0.45 μm filter membrane every 5 min, adding 0.05 mL of methanol as a quencher to the water sample. The TCH concentration in the reaction mixture was quantified using a reversed-phase C18 column (5 μm, 4.6 mm × 250 mm). Identical chromatographic conditions were maintained throughout the study to monitor TCH depletion. Analysis was performed on an Agilent 1260 HPLC system (Agilent, Santa Clara, CA, USA) using a mobile phase of methanol/acetonitrile/0.1% oxalic acid (10:25:65, *v*/*v*/*v*) at a flow rate of 1.0 mL/min. Detection was carried out at 356 nm with the column temperature set to 30 °C. All experiments were performed in triplicate independently, and the data are averaged. The removal efficiency of TCH and pseudo-first-order rate constant (k) were shown in Equations (1) and (2).

η = (C_0_ − C_t_)/C_0_ × 100% (1)

−kt = ln(C_t_/C_0_) (2)

Herein, η is the removal efficiency of TCH; C_t_ corresponds to the TCH concentration at a given reaction time, while C_0_ is the initial concentration of the TCH solution. The used catalyst was recycled and the next catalytic experiment was conducted after sufficient drying. The degradation conditions remained the same as before and the cycle was repeated three times.

### 2.4. Characterization Analysis

The surface functional groups of the prepared catalysts were characterized by a Fourier transform infrared (FTIR) spectrometer (IRTracer-100, Shimadzu, Kyoto, Japan). The crystalline structure and phase composition were analyzed via X-ray diffraction (XRD, D8 Advance, Bruker, Karlsruhe, Germany). The microscopic morphology was observed using a scanning electron microscope. The surface elemental composition and chemical bonding states of the elements were determined by X-ray photoelectron spectroscopy (XPS, ESCALAB 250XI, Thermo Fisher Scientific, Waltham, MA, USA).

## 3. Results and Discussion

### 3.1. Characterization

**Figure 1:**
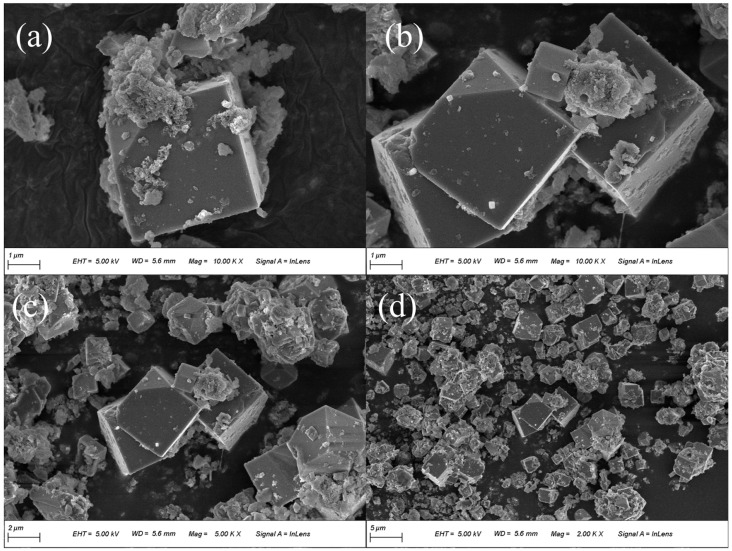
Magnified SEM image of LFC with (**a**,**b**) 10.00 K×, (**c**) 5.00 K× and (**d**) 2.00 K×.

Morphological characteristics of LFC are shown in Figure 1a–d. It can be seen that the as-prepared LFC possessed a unique composite structure composed of cubic-shaped iron-based crystals and a porous amorphous carbon matrix derived from SLS. The carbon support, assembled from aggregated nanoparticles (20–100 nm), formed a rough, wrinkled, and highly porous three-dimensional network. This structure not only provided a large specific surface area but also offered abundant channels for molecular diffusion. Combined with the XRD analysis results, these iron-containing particles were identified as likely being a mixture of Fe_3_O_4_, Fe_2_O_3_, and Fe^0^.

**Figure 2:**
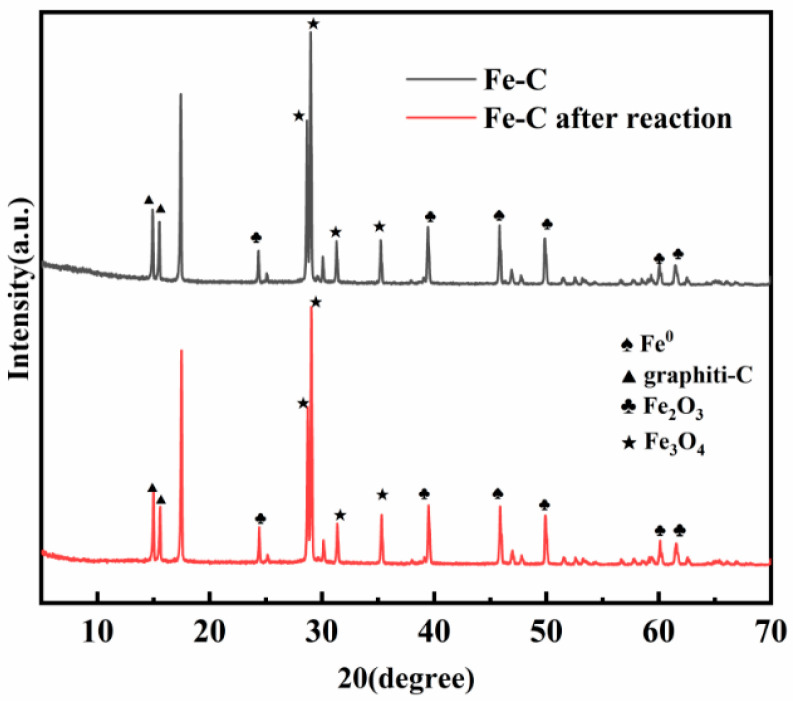
XRD patterns of LFC before and after degradation reaction.

The crystal structures of the as-prepared and the recovered after degradation of LFC were characterized by XRD, and the results are shown in Figure 2. Two broad diffraction peaks at 2θ = 14.92° and 15.53° were observed for both samples, which were assigned to the typical structures of amorphous carbon and graphitic carbon, indicating the presence of partial graphitic carbon in the material. The diffraction peaks at 24.35°, 39.44°, 49.85°, 60.06°, and 61.49° corresponded to the (012), (024), (300), (300) planes of Fe_2_O_3_. Meanwhile, the peaks at 28.66°, 29.05°, 30.07°, 31.30°, and 35.23° were indexed to the (220), (220), (220), (220), and (311) planes of Fe_3_O_4_, while those at 45.82° and 46.89° were attributed to Fe^0^ [21]. The formation of Fe^0^ was attributed to the partial reduction in FeCl_3_·6H_2_O during the pyrolysis process in the hydrothermal reactor. These results confirmed that iron species were successfully loaded onto the SLS-derived carbon matrix, forming a stable iron–carbon composite structure. It can be seen from the XRD pattern that the structure of the degraded and recycled LFC remains basically unchanged, which proved that the LFC has good stability.

**Figure 3:**
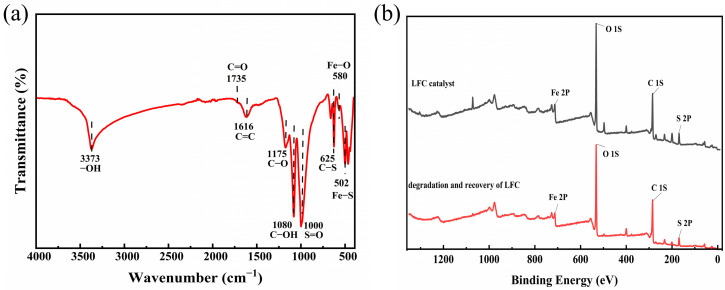
(**a**) FTIR spectrum and (**b**) XPS patterns of the LFC.

The functional groups of LFC were characterized using FTIR, as shown in Figure 3a. The broad peak that appears at a wavelength of 3373 cm^−1^ is the stretching vibration peak of -OH [22,23], the absorption peak that appears near 1735 cm^−1^ is the stretching vibration peak of alkyl C=O, the peak that appears at 1616 cm^−1^ is the stretching vibration peak of C=C [24], and the peak that appears at 1175 cm^−1^ is the stretching vibration peak of C-O. The stretching vibration peak of C-OH was at 1080 cm^−1^ [25], the peak at 1000 cm^−1^ corresponds to O=S [26], the stretching vibration peak of C-S was at 625 cm^−1^, the stretching vibration peak of Fe-O was at 580 cm^−1^ [27], and the stretching vibration peak of Fe-S was near 500 cm^−1^. These oxygen-containing functional groups could participate in non-radical reaction pathways, promoting the generation of ^1^O_2_, thereby improving the selective degradation efficiency of TCH [20].

Surface elemental compositions of the fresh LFC and the used LFC after degradation were analyzed by XPS, focusing on S 2p, C 1s, O 1s, and Fe 2p. The full survey spectra were displayed in Figure 3b, and the high-resolution XPS spectra along with the relative atomic percentages of S 2p, C 1s, O 1s, and Fe 2p are presented in the Supporting Information. As shown in Figure S1a, the S 2p spectrum of fresh LFC could be fitted into three peaks at 167.9 eV (SO_4_^2−^), 168.5 eV (S=O), and 169.8 eV (Fe-S), respectively. Compared with the fresh catalyst, the relative percentage of S 2p in the used LFC decreased, indicating that sulfur species were consumed or transformed during the catalytic reaction. Figure S1b shows the C 1s spectrum, which was deconvoluted into four peaks at 284.4 eV (C−C/C=C), 284.9 eV (C−S), 285.9 eV (C−OH), and 291.9 eV (C=O). The relative percentage of C 1s increased after the reaction, implying that surface reconstruction or chemical bond rearrangement occurred on the catalyst surface, altering the chemical environment of carbon species. In the O 1s spectrum (Figure S1c), three peaks located at 531.0 eV, 531.7 eV, and 532.5 eV were assigned to C−OH, Fe−O, and C=O, respectively. The formation of Fe−O peaks confirmed successful iron modification. After the catalytic reaction, the relative percentage of Fe−O decreased noticeably, which could be ascribed to the participation of Fe−O bonds in the activation of persulfate. As illustrated in Figure S1d, the Fe 2p spectrum was fitted with three pairs of doublets. The peaks at 711.13 eV and 724.36 eV corresponded to Fe^0^ 2p2p_3/2_ and Fe^0^ 2p_1/2_; the peaks at 713.57 eV and 726.74 eV were attributed to Fe^2+^ 2p_3/2_ and Fe^2+^ 2p_1/2_; and the peaks at 716.47 eV and 728.99 eV were assigned to Fe^3+^ 2p_3/2_ and Fe^3+^ 2p_1/2_ [28]. Both fresh and used LFC contained Fe^0^, Fe^2+^, and Fe^3+^, suggesting that iron species underwent a dynamic redox cycle during the catalytic reaction, where partial iron atoms were continuously oxidized and reduced.

### 3.2. Optimization of LFC Catalytic Performance

#### 3.2.1. Iron Source Dosage

**Figure 4:**
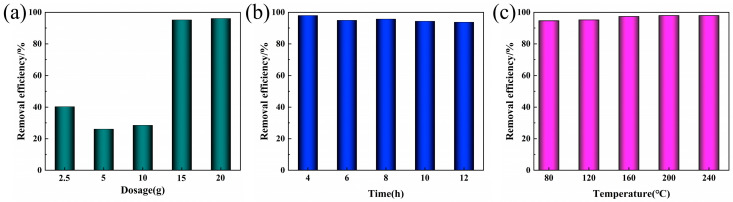
Optimum conditions for preparation of LFC with (**a**) dosage, (**b**) time, (**c**) temperature.

Five portions of 5.0 g SLS were weighed, followed by the addition of 2.5 g, 5.0 g, 10.0 g, 15.0 g, and 20.0 g of FeCl_3_·6H_2_O, respectively. The experiments were carried out according to Section 2.2, and the TCH degradation efficiency at 30 min was recorded. As shown in Figure 4a, the TCH degradation efficiency was below 45.2% when the dosage of FeCl_3_·6H_2_O was less than 15.0 g. This phenomenon could be attributed to insufficient active sites caused by the low iron dosage, resulting in low TCH degradation. When the FeCl_3_·6H_2_O dosage reached 15.0 g, the TCH degradation efficiency increased sharply to 95.2%, indicating that sufficient iron species were provided to generate reactive oxygen species (ROS), thus greatly promoting the catalytic degradation of TCH. With a further increase in FeCl_3_·6H_2_O dosage, the TCH degradation efficiency improved only slightly. The slow growth might be related to the diffusion rate of reactants and the stability of the reaction system. Therefore, the optimal dosage of FeCl_3_·6H_2_O was 15.0 g, which achieved satisfactory degradation efficiency.

#### 3.2.2. Hydrothermal Time

Five sets of samples were prepared by dissolving 5.0 g SLS and 15.0 g FeCl_3_·6H_2_O in 50 mL deionized water. The mixtures were then transferred into hydrothermal reactors and heated in an oven at 200 °C for 4 h, 6 h, 8 h, 10 h, and 12 h. All catalysts were synthesized synchronously in accordance with Section 2.2 and applied to the catalytic experiments. As depicted in Figure 4b, the TCH degradation efficiency decreased gradually with the prolongation of hydrothermal time. The highest TCH degradation rate of 98% was achieved at 4 h, suggesting that the reaction between SLS and FeCl_3_·6H_2_O reached an optimal state for the formation of the LFC. As the reaction time was further extended, the degradation efficiency declined, which could be ascribed to the reduction in accessible active sites on the LFC. Accordingly, 4 h was determined as the optimal hydrothermal time for LFC preparation.

#### 3.2.3. Hydrothermal Reaction Temperature

Five samples were prepared by dissolving 5.0 g SLS and 15.0 g FeCl_3_·6H_2_O in 50 mL deionized water. The mixtures were transferred into hydrothermal reactors and treated at different pyrolysis temperatures (80 °C, 120 °C, 160 °C, 200 °C, and 240 °C) in an oven. All catalysts were synthesized synchronously according to Section 2.2 and used for catalytic tests. As presented in Figure 4c, the TCH degradation efficiency reached 97.4% at 160 °C, indicating that the SLS exhibited favorable catalytic performance at this temperature. Additionally, further increasing the reaction temperature brought no obvious improvement in the degradation efficiency of TCH. Considering the experimental cost and operational convenience, 160 °C was selected as the optimal hydrothermal temperature.

Based on multiple experimental optimizations, the optimal conditions for preparing the LFC were 15.0 g of FeCl_3_·6H_2_O, a hydrothermal temperature of 160 °C, and a reaction time of 4 h. The LFC synthesized under these conditions exhibited the best catalytic performance, providing a favorable catalytic material for the degradation experiments of organic pollutants.

### 3.3. Degradation of TCH by Different Catalytic Systems

#### 3.3.1. Catalytic Performance of LFC

**Figure 5:**
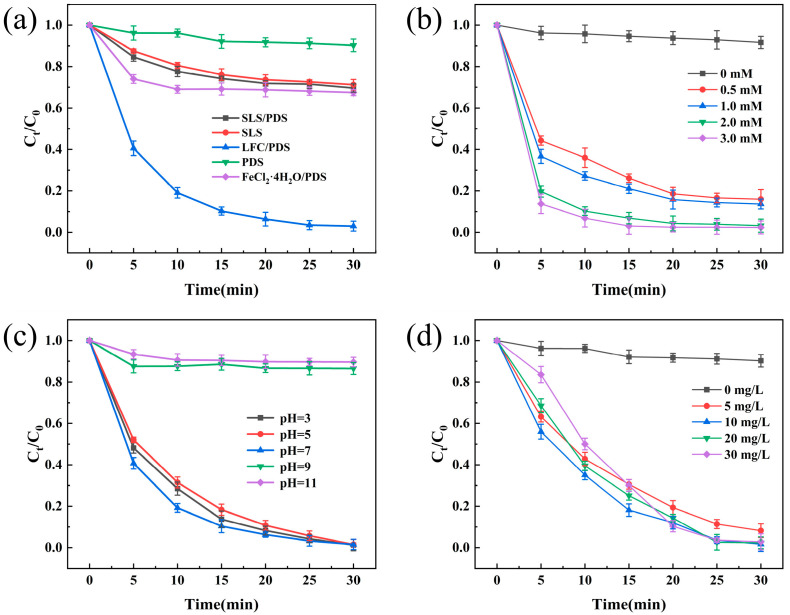
Degradation curves of LFC with (**a**) different catalytic systems, (**b**) different PDS dosages, (**c**) different initial pH values, and (**d**) different catalyst dosages. General experimental condition: C_TCH_ = 50 mg/L, C_PDS_ = 2.0 mM, C_catalyst_ = 10 mg/L, initial pH = 7.0.

The effects of different catalytic systems on TCH degradation efficiency were evaluated, including SLS/PDS, LFC/PDS, PDS alone, and FeCl_2_·4H_2_O/PDS. As shown in Figure 5a, PDS alone could barely degrade TCH within 30 min, indicating very weak self-activation ability toward TCH. The SLS/PDS and FeCl_2_·4H_2_O/PDS systems only achieved limited TCH degradation, with removal efficiencies of 30.4% and 32.3%, respectively. In contrast, the LFC/PDS and activated LFC/PDS systems efficiently degraded TCH, reaching degradation efficiencies of 97.7% and 97%. These results demonstrated that LFC could effectively activate PDS and significantly improve its activation performance. The enhanced degradation capacity could be attributed to the efficient activation of PDS by LFC, which promoted the decomposition of PDS into ROS and further accelerated TCH degradation. By comparison, SLS and FeCl_2_·4H_2_O could also promote PDS activation, but the catalytic performances were much weaker than that of LFC.

#### 3.3.2. Effect of PDS Dosages

To evaluate the effect of PDS concentration on TCH degradation, a series of experiments was performed at five different initial concentrations. As shown in Figure 5b, the degradation efficiency of TCH strongly depended on the PDS concentration. The degradation rate of TCH was 8.4% when no PDS was added and the removal efficiency did not exceed 90% within 30 min at 1.0 mM of PDS. When the PDS concentration increased to 2.0 mM, the removal efficiency was significantly improved to 96.8% within 30 min, and the kobs value was significantly increased (Figure S2a), confirming that a higher PDS concentration accelerated the generation of ROS and thus boosted the catalytic degradation rate of TCH. With a further increase in PDS dosage to 3.0 mM, the removal efficiency of TCH showed only a slight improvement, and only a small increment in kobs was observed, indicating that excessive PDS cannot be fully activated by the limited active sites on LFC and may even induce self-quenching of reactive species. These results indicated that the optimal PDS dosage under the experimental conditions was 2.0 mM.

#### 3.3.3. Effect of pH

As shown in Figure 5c, the effect of initial pH on TCH removal in the LFC/PDS system was investigated. Within the pH range of 3–7, the TCH removal efficiency exceeded 98.7% within 30 min. Meanwhile, the kobs remained at a high level in the acidic or neutral conditions (Figure S2b). However, the TCH removal efficiency dropped sharply to below 15% when pH exceeded 7, confirming that acidic-to-neutral media favor PDS activation and ROS generation. In addition, the alkaline condition could accelerate the reaction of sulfate radical (SO_4_^−^·) with OH^−^ and convert it to hydroxyl radical (·OH) with lower oxidation potential, accompanied by the hydrolysis of iron species into Fe(OH)_3_ precipitates that block active sites. Since near-complete removal could be achieved at the original pH of 7 without additional adjustment, pH 7 was selected as the optimal condition for subsequent experiments.

#### 3.3.4. Effect of Catalyst Dosages

Catalyst dosage serves as a critical operational parameter that exerts a substantial influence on PDS activation efficiency and the resulting TCH degradation performance. As depicted in Figure 5d, the control system without LFC addition achieved only 9.8% TCH removal within 30 min, reflecting the inherently weak oxidation capacity of PDS alone for TCH elimination. When the LFC dosage was elevated from 5 mg/L to 10 mg/L, the TCH degradation efficiency was markedly enhanced. At an LFC dosage of 10 mg/L, the TCH removal efficiency reached 98.4% after 30 min of reaction, and the corresponding observed k_obs_ attained its maximum value simultaneously (Figure S2c). Nevertheless, further increases in LFC dosage to 20 mg/L and 30 mg/L failed to yield continuous improvement in degradation performance; instead, the 30 min TCH removal efficiencies declined slightly to 97.7% and 97.2%, respectively, coupled with a marginal decrease in k_obs_ values. The excessive LFC contributed marginally to the degradation efficiency and even weakened the degradation effect, likely because the activated PDS had already been saturated with ROS and the additional catalyst might even trigger ROS self-quenching. Therefore, considering both the high removal performance and cost-effectiveness, a catalyst dosage of 10 mg/L was identified as the optimal condition.

### 3.4. Stability of LFC

**Figure 6:**
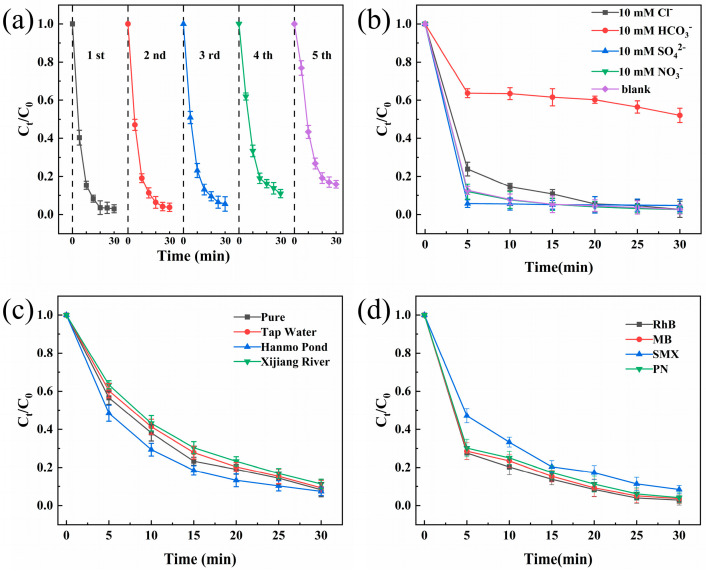
Effects of (**a**) reusability experiments, (**b**) anions, (**c**) water matrices on TCH degradation, and (**d**) effects of different organic pollutants in the LFC/PDS system. General experimental condition: C_TCH_ = 50 mg/L, C_PDS_ = 2.0 mM, C_catalyst_ = 10 mg/L, initial pH = 7.0.

The long-term operational feasibility of LFC was systematically evaluated via five consecutive recycling experiments under identical experimental conditions, and the corresponding results were displayed in Figure 6a. Unlike many conventional iron-based catalysts that tend to deactivate rapidly, LFC still maintained a TCH removal efficiency above 85% after 5 cycles. Furthermore, ICP determined the concentration of iron ions leached into the solution after the first reaction cycle to be 2.87 mg/L. The amount of leached iron was markedly lower than the total iron content of the LFC, which accounts for 3.7% of the catalyst mass. Although a moderate decline in catalytic activity was observed after repeated usage, no severe and abrupt deactivation phenomenon occurred. Consequently, the LFC achieved a good balance between durable stability and satisfactory catalytic activity. The cyclic experimental results confirmed the good application potential of this catalyst in practical wastewater treatment.

Compared with previously reported iron-based carbon catalysts for TCH degradation (Table 1), the LFC in this work achieved 100% TCH removal in only 30 min at a low dosage of 10 mg/L, outperforming most systems in both efficiency and reaction rate. Moreover, LFC maintained a removal efficiency of 84.1% after five cycles, demonstrating superior reusability and stability compared with many reported materials and making it a promising candidate for practical antibiotic wastewater treatment.

### 3.5. Practicability of LFC

Common anions, such as HCO_3_^−^, Cl^−^, NO_3_^−^, and SO_4_^2−^, which widely exist in actual wastewater, may interfere with reactive species during TCH degradation. Herein, the effects of different coexisting anions on TCH degradation in LFC/PDS system were explored, and the results are displayed in Figure 6b. Except for HCO_3_^−^, other anions exerted negligible impacts on the TCH removal, and the degradation efficiencies all remained above 95% within 30 min. The addition of Cl^−^, NO_3_^−^, and SO_4_^2−^ barely changed the solution pH, which accounted for their slight inhibitory effect on TCH degradation. By contrast, the introduction of HCO_3_^−^ sharply reduced the TCH degradation efficiency to 47.9% within 30 min. This phenomenon was mainly because HCO_3_^−^ rapidly increased the solution pH, and the elevated alkaline environment was unfavorable for TCH removal. Additionally, the LFC/PDS system maintained high TCH removal efficiency across various real water matrices, including deionized water, tap water, pond water, and river water. Although coexisting organic matter and background components slightly reduced the degradation rate in natural waters, the removal efficiency consistently remained above 88% (Figure 6c), demonstrating its strong anti-interference capability and potential for practical application. Overall, under identical experimental conditions, the LFC/PDS system achieved removal rates exceeding 90% within 30 min for four representative organic contaminants, namely RhB, MB, SMX, and PN, when tested individually (Figure 6d). These results confirm that the LFC/PDS system maintains stable degradation efficiency across pollutants with varying molecular structures, underscoring its versatility in treating various contaminants for environmental remediation.

### 3.6. Degradation Mechanism of LFC/PDS System

#### 3.6.1. Quenching Experiment and Ecotoxicological Assessment

**Figure 7:**
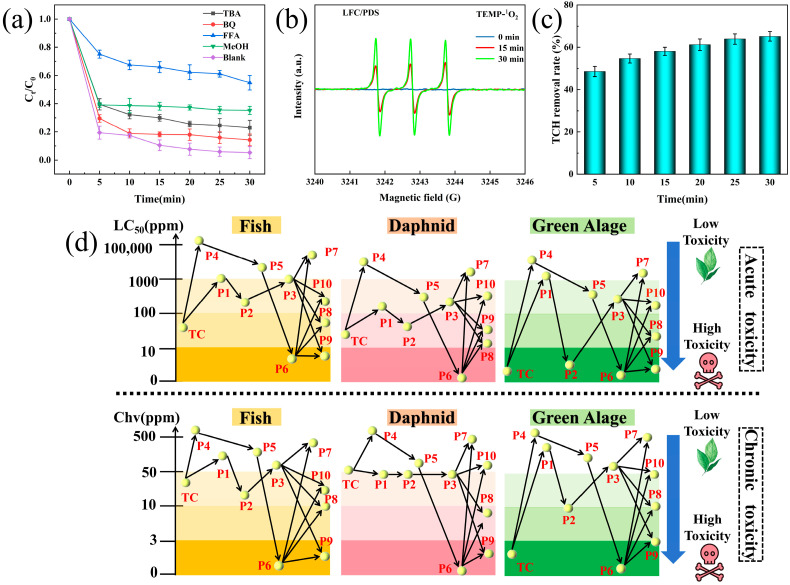
(**a**) Degradation efficiency of TCH after the quenching experiment, (**b**) EPR spectrum of TEMP-^1^O_2_, (**c**) the removal effect of TOC, and (**d**) ecotoxicological evaluation of TCH intermediates in the LFC/PDS system.

To identify the key reactive species involved in TCH degradation, quenching experiments were carried out using 10 mM TBA, BQ, FFA, and MeOH as four chemical scavengers [34]. The experimental procedures were the same as degradation experiment of TCH, except that the designated scavenger was added prior to PDS. Specifically, MeOH scavenged both SO_4_^−^· and ·OH, TBA quenched ·OH, BQ eliminated ·O_2_^−^, and FFA captured ^1^O_2_. As shown in Figure 7a, the TCH degradation efficiency reached 94.7% within 30 min in the absence of scavengers. The degradation efficiency decreased to 64.8% and 77.1% after adding MeOH and TBA, indicating that both ·OH and SO_4_^−^· were generated during TCH degradation. When BQ and FFA were introduced, the TCH degradation efficiencies were 85.8% and 45.2% within 30 min, demonstrating that ^1^O_2_ served as a crucial reactive species over the non-radical pathway. In addition, EPR spectroscopy with TEMP as the spin-trapping agent was performed, and the corresponding spectral results are displayed in Figure 7b. No characteristic triplet signal assigned to the TEMP-^1^O_2_ adduct was detected at 0 min; however, distinct triplet peaks emerged at 15 min and intensified at 30 min, visually confirming the continuous accumulation of ^1^O_2_ with prolonged catalytic reaction time. The remarkable suppression of TCH removal efficiency by FFA, combined with the time-dependent enhancement of TEMP-^1^O_2_ EPR signals, collectively corroborates that ^1^O_2_ was the dominant non-radical active species responsible for TCH elimination in the LFC/PDS system. Furthermore, these results confirm that ^1^O_2_ generation relied on the catalytic activation of PDS by LFC, rather than spontaneous production from LFC or PDS alone. As shown in Figure 7c, demonstrates that the catalytic system also exhibited favorable total organic carbon (TOC) removal performance, with a TOC removal efficiency of 65.2% achieved after 30 min of reaction. These results further confirm that the LFC/PDS system possesses excellent catalytic activity and achieves a certain degree of mineralization toward TCH.

To further evaluate the environmental implications of the degradation process, the ecotoxicity profiles of TCH and its identified intermediates (P1-P10) were assessed. As illustrated in Figure 7d, compared with the parent TCH, most degradation intermediates exhibit significantly attenuated toxicity. Among, intermediate P6 displayed the highest acute and chronic toxicity across all tested species, with the lethal concentration 50% (LC_50_) for fish and the chronic value (Chv) for daphnid both below 10 ppm. Next, intermediate P4 and P7 exhibited low toxicity to fish, daphnid and green alage. The remaining intermediates posed relatively limited ecological risks. Collectively, these results confirmed that the LFC/PDS system effectively mitigated the long-term ecological risk of TCH. In addition, coupled with the 65.2% TOC removal efficiency, the pronounced capacity of the system to transform TCH into low-toxicity intermediates validates its superior degradation performance.

#### 3.6.2. Removal Mechanism

High-performance liquid chromatography–tandem mass spectrometry (HPLC-MS/MS) was employed to detect the degradation products of TCH in the LFC/PDS system, and a series of intermediate products with mass-to-charge ratios (*m/z*) of 426, 337, 307, 247, 226, 214, 168, 142, 136 and 98 were identified. The possible degradation pathways of TCH and the HPLC profiles of TCH degradation in the LFC/PDS system are shown in Figures S3 and S4. Pathway 1 involved the elimination of the methyl group on the second ring of the tetracycline molecule and the release of a water molecule to form intermediate P1, followed by fourth-ring opening cleavage, decarbonylation and gradual hydroxyl loss of P1 to generate intermediates P2 and P3 sequentially [35,36]. Pathway 2 featured the detachment of two methyl groups on the fourth ring of the tetracycline molecule to yield intermediate P4, after which P4 underwent ring opening and hydroxylation to produce intermediate P5, and P5 was further subjected to ring cleavage to form intermediate P6 [36]. Ultimately, the low-molecular-weight organic intermediates P6, P7, P8, P9 and P10 were further oxidized and decomposed into CO_2_, H_2_O and other small-molecule products under the oxidative action of ^1^O_2_,·OH, and SO_4_^−^· in the system.

**Figure 8:**
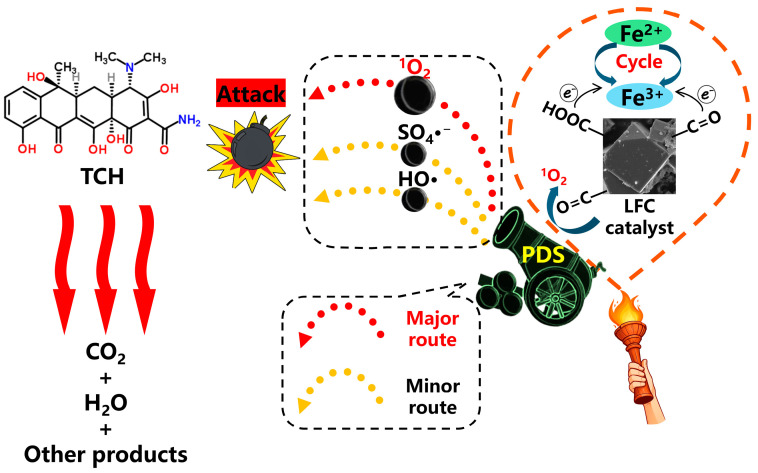
Mechanism of LFC-activated PDS for TCH degradation.

Based on the results of quenching experiments and material characterizations, the possible degradation mechanism of LFC/PDS system to degrade TCH was proposed as shown in Figure 8. Specifically, Fe^0^, Fe^2+^ and Fe^3+^ in LFC could activate PDS effectively to form SO_4_^−^ (Equations (3)–(5)) [37,38]. At the same time, excellent iron cycle further promoted ROS production and increased TCH degradation rate (Equation (6)) [37,39]. The generated SO_4_^−^· reacted with water to form ·OH, which participated in the degradation reaction (Equation (7)). In addition, the electron transfer in LFC activated PDS and could further generate free radicals (Equations (8) and (9)) [40,41]. Then, the generated ·O_2_^−^ radicals were further oxidized to ^1^O_2_. This arises from the nucleophilic nature of O_2_^−^ and the electron-rich characteristic of TCH, which hinder their direct interaction. Consequently, ·O_2_^−^ tended to react with H_2_O or ·OH, thereby facilitating the formation of ^1^O_2_ (Equations (10) and (11)) [42]. Among them, the oxygen-containing functional groups (OFG) present in LFC can also be used as the generation of non-radical active sites ^1^O_2_ of biochar-activated PDS to further enhance the degradation effect (Equation (12)) [43]. As an electrophilic reactive oxygen species with high selectivity, ^1^O_2_ preferentially attacks the electron-rich regions of TCH molecules [44,45]. TCH gradually decomposes into small molecular organic intermediates with lower molecular weight under oxidation of ^1^O_2_, SO_4_^−^· and ·OH [46]. In conclusion, the LFC/PDS system followed a hybrid radical/non-radical oxidation process. The synergistic effect of iron redox cycling and OFG on the catalyst surface promoted the directional generation of ^1^O_2_, endowing the system with excellent catalytic activity, selectivity, and anti-interference ability [47,48].

Fe^0^ + 2S_2_O_8_^2−^→Fe^2+^ + 2·SO_4_^−^ + 2SO_4_^2−^ (3)

Fe^3+^ + S_2_O_8_^2−^→Fe^2+^ + S_2_O_8_^−^ (4)

Fe^2+^ + S_2_O_8_^2−^→Fe^3+^ + SO_4_^−^·+ SO_4_^2−^ (5)

Fe^0^ + Fe^3+^→3Fe^2+^ (6)

SO_4_^−^·+ H_2_O→H^+^ +·OH + SO_4_^2−^ (7)

S_2_O_8_^2−^ + 2H_2_O→·HO_2_^−^ + 3H^+^ + 2SO_4_^2−^ (8)

S_2_O_8_^2−^ + HO_2_^−^→·O_2_^−^ + H^+^ + SO_4_^2−^ + SO_4_^−^ (9)

O_2_^−^ + 2H_2_O→^1^O_2_ + H_2_O_2_ + 2OH^−^ (10)

O_2_^−^ +·OH→^1^O_2_ + OH^−^ (11)

S_2_O_8_^2−^ + OFG + 2H_2_O→1O_2_ + 2SO_4_^2−^ + 4H^+^ (12)

## 4. Conclusions

A lignosulfonate-derived iron–carbon composite catalyst was successfully prepared via the hydrothermal method, and its catalytic performance for PDS activation to degrade TCH was systematically investigated. The results of characterization confirmed that LFC consisted of iron-based crystals uniformly distributed on a porous amorphous carbon matrix, which provided abundant active sites and accelerated mass transfer. Under the conditions of adding 10 mg/L LFC, 2.0 mM PDS and initial pH 7, the TCH removal efficiency was achieved nearly 100% within 30 min. The LFC/PDS system exhibited high efficiency over a broad pH range of 3–7, strong anti-interference capacity, and good universality for degrading various organic pollutants. After five consecutive cycles, the TCH removal efficiency still retained over 84.1% in LFC/PDS system, demonstrating that the LFC had excellent structural robustness and reusability. Notably, the TCH degradation process was dominated by a non-radical pathway with ^1^O_2_, assisted by a minor radical pathway involving SO_4_^−^· and ·OH, in which the synergistic effect of iron redox cycling and oxygen-containing functional groups played a critical role. This research provides a low-cost, eco-friendly, and efficient catalyst for antibiotic wastewater treatment and offers a promising strategy for the value-added utilization of lignin biomass in advanced oxidation processes.

## Figures and Tables

**Table 1:** Comparison of TCH degradation efficiency and catalyst stability for PDS-activated systems.

Catalysts	Conditions	Time (min)	Efficiency (%)	Stability	Reusability Effect (%)	Ref
Cr–Fe/BC	[TCH] = 50 mg/L, [catalyst] = 0.03 g/L, [PDS] = 2.0 mM, pH = 7.0	60	99.5	8th run	50	[29]
BET/Fe-C/PDS	[TCH] = 10 mg/L, [catalyst] = 80 mg/L, [PDS] = 2.0 mM, pH = 5.56	40	98.62	-	-	[30]
SSBC	[TCH] = 10 mg/L, [catalyst] = 0.6 g/L, [PDS] = 4.0 mM, pH = 5.0	180	87.4	4th run	69.4	[31]
MMBC	[TCH] = 10 mg/L, [catalyst] = 0.5 g/L, [PDS] = 2.0 mM, pH = 7.0	60	93.8	3th run	60.0	[32]
NMPC	[TCH] = 50 mg/L, [catalyst] = 0.25 g/L, [PDS] = 2.0 mM, pH = 6.0	120	99.8	5th run	92.4	[33]
LFC	[TCH] = 50 mg/L, [catalyst] = 10 mg/L, [PDS] = 2.0 mM, pH = 7.0	30	100	5th run	84.1	This work

## Data Availability

The original contributions presented in this study are included in the article/Supplementary Material. Further inquiries can be directed to the corresponding authors.

## Supplementary Materials

The following supporting information can be downloaded at https://www.mdpi.com/article/10.3390/toxics14070606/s1: Figure S1 XPS of LFC catalyst and (a) S 2p, (b) C 1s, (c) O 1s and (d) Fe 2p of LFC recovered by degradation. Figure S2. Degradation kinetic curves of LFC under different conditions with (a) Dosage of PDS (b) Initial pH (c) Catalyst dose. Figure S3 Proposed degradation pathway and intermediates generation during TCH degradation by LFC/PDS system. Figure S4 High performance liquid chromatography analysis of TCH degradation by LFC/PDS.

## Abbreviations

The following abbreviations are used in this manuscript:

(PDS)	Peroxydisulfate
(TCH)	Tetracycline Hydrochloride
(^1^O_2_)	Singlet Oxygen
(ARGs)	Antibiotic Resistance Genes
(ARB)	Antibiotic-Resistant Bacteria
(PS-AOPs)	Persulfate-based Advanced Oxidation Processes
(SO_4_^−^·)	sulfate radicals
(·OH)	hydroxyl radicals
(SLS)	Sodium Lignosulfonate
(LFC)	Lignosulfonate-Fe Carbonized Catalyst
(MeOH)	Methanol
(FFA)	Furfuryl alcohol
(TBA)	tert-butanol
(BQ)	p-benzoquinone
(RhB)	Rhodamine B
(MB)	Methylene Blue
(SMX)	Sulfamethoxazole
(PN)	Phenol
(XRD)	X-ray diffraction
(ROS)	reactive Oxygen Species
(TOC)	total organic carbon
(LC_50_)	lethal concentration 50%
(Chv)	chronic value
(HPLC-MS/MS)	High-performance liquid chromatography–tandem mass spectrometry
(OFG)	oxygen-containing functional groups
