# Supplementary material for: Peroxydisulfate Activation by Lignosulfonate-Derived Iron–Carbon Catalyst for Tetracycline Hydrochloride Removal: Contributions of 1O2 and Iron Cycle
Source: Toxics. 2026 Jul 11;14(7):606. doi: 10.3390/toxics14070606 (PMC13418887; doi:10.3390/toxics14070606)
Supplement: Supplementary file 1 [file toxics-14-00606-s001.zip › toxics-4413181-supplementary.pdf]

Supporting Information for

Peroxydisulfate activation by lignosulfonate-derived iron-carbon catalyst for tetracycline hydrochloride removal: Contributions of $^1O_2$ and Fe cycle

This supporting information include 2 figures (Figure S1 to S2).

Figures:

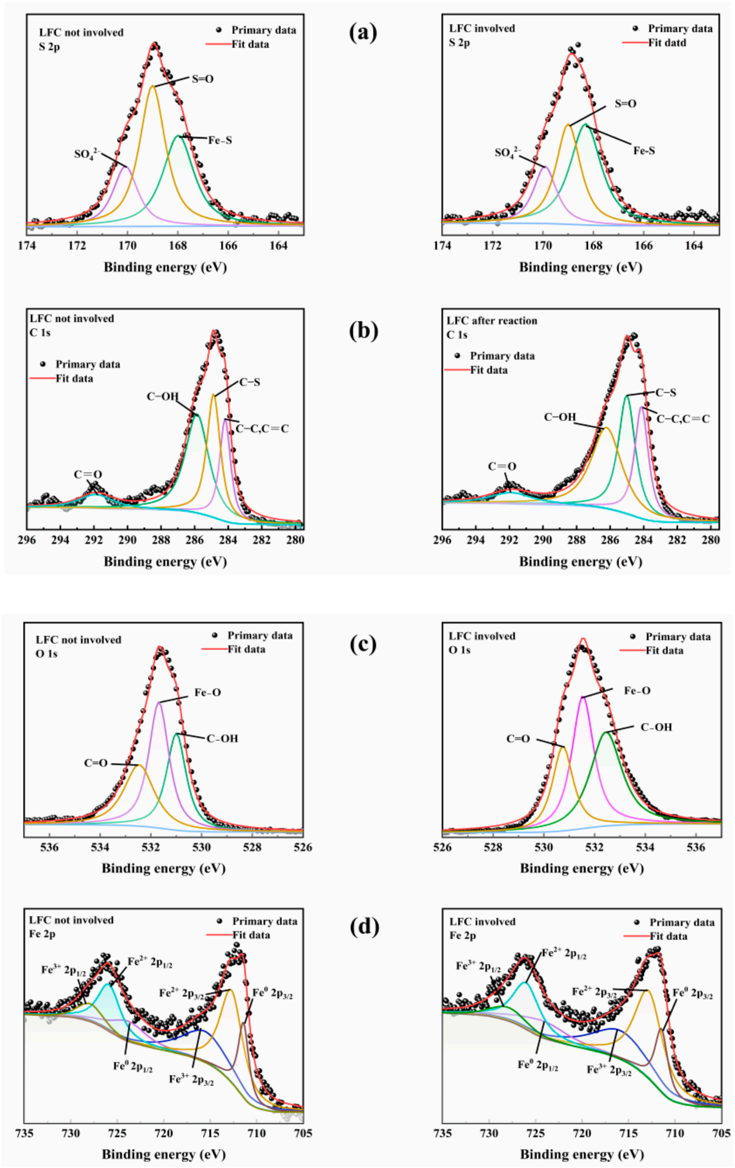

Figure S1. XPS of LFC catalyst and (a) S 2p, (b) C 1s, (c) O 1s and (d) Fe 2p of LFC recovered by degradation.

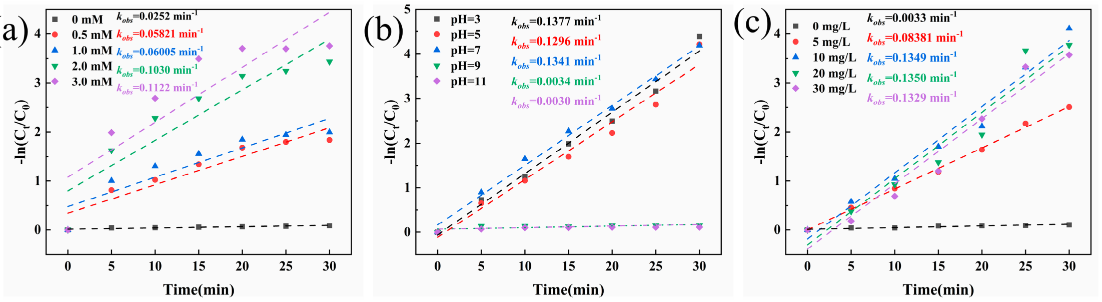

Figure S2. Degradation kinetic curves of LFC under different conditions with (a) Dosage of PDS (b) Initial pH (c) Catalyst dose

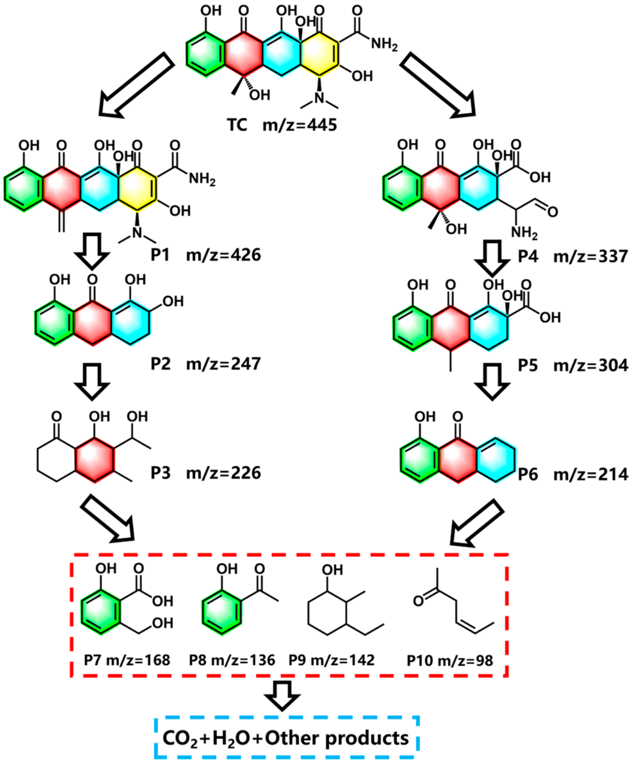

Figure S3. Proposed degradation pathway and intermediates generation during TCH degradation by LFC/PDS system

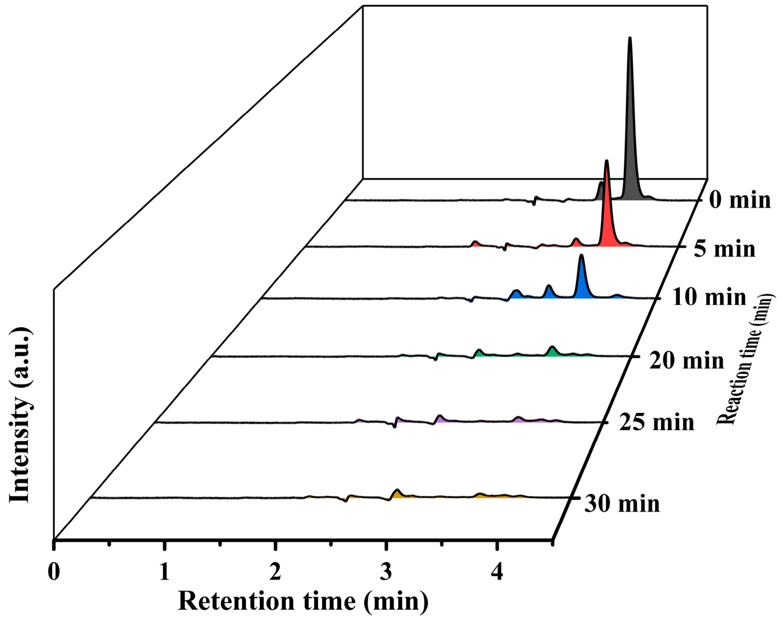

Figure S4. High performance liquid chromatography analysis of TCH degradation by LFC/PDS
